# Interpreting Continuous Glucose Monitoring Through Metabolic Physiology: A Framework for Precision Nutrition in Type 2 Diabetes

**DOI:** 10.3390/nu18152578

**Published:** 2026-08-06

**Authors:** Jitendra D. Belani

**Affiliations:** Jefferson College of Pharmacy, Thomas Jefferson University, 1020 Locust Street, Philadelphia, PA 19107, USA; jitendra.belani@jefferson.edu; Tel.: +1-215-503-6867

**Keywords:** continuous glucose monitoring, glycemic variability, precision nutrition, glycemic phenotypes, type 2 diabetes, metabolic physiology

## Abstract

**Background**: Type 2 diabetes is characterized by substantial interindividual variability in postprandial glucose responses, yet continuous glucose monitoring (CGM)-derived glucose patterns are commonly described using quantitative metrics without sufficient consideration of the physiological mechanisms that generate them. **Objective**: To develop a physiology-based framework for interpreting CGM-derived glycemic phenotypes in relation to dietary exposures and individual metabolic characteristics, thereby supporting precision nutrition in adults with type 2 diabetes. **Methods**: A structured narrative review of the literature was conducted using PubMed to identify studies examining CGM, postprandial glucose regulation, dietary exposures, metabolic physiology, and precision nutrition. Evidence was synthesized within a predefined conceptual framework linking dietary exposures, underlying physiology, individual metabolic characteristics, CGM-derived glycemic phenotypes, and their clinical interpretation. **Results**: Postprandial glucose responses reflect coordinated interactions among nutrient digestion and absorption, hormonal regulation, hepatic glucose production, peripheral glucose disposal, and individual metabolic characteristics rather than dietary carbohydrate exposure alone. The proposed framework interprets recurring CGM-derived glucose patterns as physiologically meaningful glycemic phenotypes, including characteristic patterns of postprandial excursion, recovery, baseline glycemia, and glycemic variability. This framework provides a structured approach for physiology-informed interpretation of CGM observations and individualized nutritional assessment. Although direct evidence supporting phenotype-guided nutritional interventions and long-term clinical benefit remains limited, emerging computational approaches may further strengthen this interpretive process by integrating CGM with complementary biological and behavioral data. **Conclusions**: Rather than viewing CGM solely as a technology for measuring glucose, the proposed framework positions it as a physiological lens through which dietary exposures, metabolic regulation, and individual metabolic characteristics can be integrated to support precision nutrition in type 2 diabetes.

## 1. Introduction

Type 2 diabetes mellitus is characterized not only by chronic hyperglycemia but also by substantial heterogeneity in the way individuals respond to food. Even when consuming similar meals, people with comparable glycated hemoglobin levels may exhibit significantly different postprandial glucose excursions, recovery trajectories, and patterns of glycemic variability, generating distinct glycemic phenotypes [1,2,3]. These differences reflect complex interactions between dietary composition and the underlying metabolic characteristics of the individual, including insulin sensitivity, beta-cell function, hepatic glucose regulation, body composition, physical activity, sleep, and circadian biology [2,3]. Understanding the biological basis of this heterogeneity has become increasingly important as nutritional management shifts from standardized dietary recommendations toward more individualized approaches.

Historically, long-term glycemic control has been evaluated primarily using glycated hemoglobin (HbA1c), a robust marker of average glucose exposure over approximately three months [4,5]. Although HbA1c remains indispensable for diagnosis, risk stratification, and treatment monitoring, it provides limited insight into the dynamic physiological responses that occur throughout the day [6]. Individuals with similar HbA1c values may experience substantially different postprandial glucose excursions, glycemic variability, and periods of hyperglycemia or hypoglycemia, all of which contribute to metabolic stress and may influence clinical outcomes. Continuous glucose monitoring has transformed the assessment of glycemic control by providing high-resolution measurements of glucose fluctuations under free-living conditions, thereby revealing dimensions of glucose regulation that are not captured by HbA1c alone [7,8].

Foundational studies of individualized glycemic responses first highlighted an important biological question: why do individuals consuming similar foods often exhibit markedly different glycemic responses? [1,2] Evidence accumulated over the past decade suggests that postprandial glucose excursions cannot be explained by dietary carbohydrate content alone [9,10]. Rather, they emerge from interactions between macronutrient composition, meal structure, gastrointestinal physiology, hormonal regulation, insulin action, tissue-specific glucose uptake and disposal, and individual metabolic characteristics [11,12,13]. Although these foundational studies established substantial interindividual variability in glycemic responses, they did not directly validate physiology-based CGM phenotype classifications in adults with type 2 diabetes. These interacting processes produce distinct glycemic phenotypes that become observable through continuous glucose monitoring, yet the physiological interpretation of these phenotypes remains incompletely defined [14,15].

Although numerous reviews have examined continuous glucose monitoring, dietary interventions, glycemic variability, and precision nutrition, these topics have generally been explored from separate clinical, nutritional, or technological perspectives [6,16,17]. As a result, CGM-derived outcomes are frequently described in terms of glucose metrics rather than interpreted through the underlying physiological processes that generate them [15,18]. Existing reviews provide valuable syntheses of dietary strategies, glucose monitoring technologies, and metabolic pathways, yet relatively few integrate these domains into a coherent framework that explains how dietary exposures interact with individual metabolic characteristics to produce the glycemic phenotypes observed through CGM [17]. Consequently, the physiological significance of many CGM-derived patterns remains incompletely understood, limiting their interpretation and application to individualized nutritional care.

**Figure 1:**
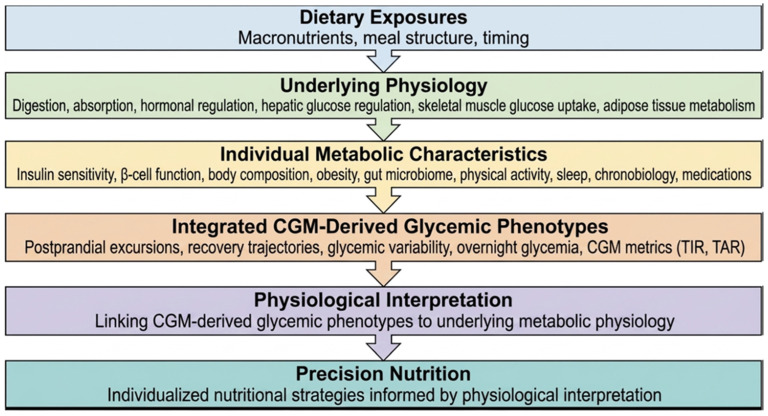
Conceptual framework linking dietary exposures to CGM-derived glycemic phenotypes and precision nutrition in type 2 diabetes. Abbreviations: CGM, continuous glucose monitoring; TIR, time in range; TAR, time above range; β-cell, pancreatic beta cell. Created in BioRender. Belani, Jitendra D. (2026) (https://app.biorender.com/illustrations/canvas-beta/6a733b6ad45a540ebc3d7688, accessed on 8 May 2026).

The objective of this review is to establish a physiology-based framework for interpreting CGM-derived glucose responses to support precision nutrition in adults with type 2 diabetes [16,17]. This review therefore addresses the following central question: How do macronutrient composition and individual metabolic characteristics shape CGM-derived postprandial glucose responses in adults with type 2 diabetes, and how can these insights support precision nutrition? To address this question, the review synthesizes evidence across dietary physiology, metabolic regulation, individual modifiers of glycemic response, and emerging precision nutrition strategies [2]. Rather than viewing continuous glucose monitoring solely as a monitoring technology, the proposed framework positions CGM as a physiological lens through which dietary exposures, metabolic regulation, and individual biological characteristics can be integrated to explain glycemic phenotypes and inform precision nutrition. This framework serves as the conceptual foundation for the sections that follow. Its overall structure is illustrated in Figure 1, while Table 1 summarizes the physiological interpretation of CGM-derived glycemic phenotypes and their implications for precision nutrition.

## 2. Review Methodology

This review was conducted as a structured narrative review [41,42] examining how dietary macronutrient composition and individual metabolic characteristics shape continuous glucose monitoring (CGM)-derived postprandial glucose responses in adults with type 2 diabetes, and how these responses can be interpreted to support precision nutrition. Rather than providing a descriptive overview of CGM, nutrition, or metabolic physiology as independent topics, the review was developed around a predefined physiological framework linking dietary exposures, underlying biological mechanisms, CGM-derived glycemic phenotypes, and their clinical interpretation.

A structured literature search was performed using PubMed to identify studies published between January 2020 and July 2026 that examined type 2 diabetes, continuous glucose monitoring, postprandial glucose responses, dietary macronutrients, meal timing, glycemic variability, and precision nutrition. Medical Subject Headings [43] and title or abstract search terms were combined to maximize retrieval of both indexed and recently published studies. The search strategy combined these controlled vocabulary terms with free-text terms including “continuous glucose monitoring,” “type 2 diabetes,” “postprandial glucose,” “glycemic variability,” “precision nutrition,” “dietary carbohydrates,” “meal timing,” “insulin resistance,” and related concepts. Landmark studies published before 2020 were included when they represented foundational evidence for CGM, metabolic physiology, or nutritional interventions relevant to the proposed framework. Additional targeted searches and reference list screening [44] were performed to identify studies addressing individual metabolic modifiers, including insulin resistance, obesity, body composition, physical activity, sleep, chronobiology, the gut microbiome, and emerging precision nutrition technologies.

Retrieved studies were screened for relevance to the predefined review question and categorized according to their contribution to the conceptual framework. Priority was given to randomized controlled trials, controlled feeding studies, crossover trials, prospective cohort studies, systematic reviews, and meta-analyses conducted in adults with type 2 diabetes [45,46]. Mechanistic investigations and carefully selected studies involving obesity, insulin resistance, prediabetes, or metabolically healthy adults were included when they provided essential biological context for interpreting CGM-derived glycemic responses. Clinical studies were prioritized when they included CGM-derived outcomes. Studies without CGM were selectively retained when they provided foundational physiological or mechanistic evidence necessary for interpreting postprandial glucose responses and CGM-derived glycemic phenotypes.

Because this review proposes a conceptual physiological framework rather than a quantitative evidence synthesis, the maturity of the evidence was interpreted qualitatively rather than through formal certainty grading [46]. Findings supported by consistent physiological mechanisms and multiple high-quality clinical investigations were considered established, whereas evidence derived from exploratory studies, emerging technologies, or limited clinical datasets was interpreted more cautiously. Throughout the review, emphasis was placed on integrating mechanistic and clinical evidence to explain how physiological processes shape CGM-derived glycemic phenotypes and how these phenotypes may inform individualized nutritional strategies.

## 3. Why Do Identical Meals Produce Different Glucose Responses?

### 3.1. Interindividual Variability in Postprandial Glycemic Responses

The traditional view that individuals consuming similar meals will exhibit comparable glycemic responses has been challenged by evidence demonstrating substantial interindividual variability in postprandial glucose regulation [1,2,47]. Even among adults with similar HbA1c levels, age, body mass index, or diabetes duration, CGM frequently reveals substantially different glucose excursions, recovery trajectories, and patterns of glycemic variability following comparable dietary exposures [7,14]. These observations suggest that postprandial glucose responses reflect considerably more than carbohydrate quantity alone.

Recent investigations have shown that dietary macronutrient composition interacts with multiple physiological processes to shape postprandial glycemic responses [9,12,48]. Digestion, gastric emptying, incretin secretion, insulin release, hepatic glucose production, peripheral glucose disposal, and tissue-specific insulin sensitivity collectively influence the magnitude and temporal characteristics of glucose excursions. Consequently, identical meals may generate substantially different CGM-derived glycemic phenotypes across individuals because the underlying physiological responses differ.

Large observational studies and controlled feeding investigations have consistently reported substantial interindividual variability in glycemic responses, reflecting measurable biological heterogeneity. Individual metabolic characteristics, including insulin sensitivity, beta-cell function, body composition, habitual physical activity, sleep, circadian regulation, gut microbial composition, medication use, and genetic background, each contribute to the variability observed following similar dietary challenges [1,2]. Together, these findings indicate that postprandial glucose responses represent integrated physiological phenomena rather than simple reflections of nutrient intake.

The recurring patterns observed across individuals suggest that CGM responses may be viewed not simply as isolated glucose measurements, but as distinct glycemic phenotypes. These phenotypes encompass characteristic patterns of postprandial excursions, recovery trajectories, baseline glycemia, and glycemic variability that emerge from the interaction between dietary exposures and underlying metabolic physiology. Interpreting these phenotypes provides a framework for understanding biological heterogeneity and for developing individualized nutritional strategies.

### 3.2. Intraindividual Variability and the Reproducibility of CGM Responses

Although interindividual variability is well recognized, glycemic responses are also variable within the same individual [2,3]. Identical meals consumed on different days may produce similar overall response patterns while differing in peak glucose, recovery time, or measures of glycemic variability. This observation highlights that postprandial glucose regulation is influenced not only by relatively stable physiological characteristics but also by dynamic factors that fluctuate from day-to-day.

Recent reproducibility studies suggest that some CGM-derived metrics demonstrate substantially greater stability than others [49,50]. Mean postprandial glucose and overall excursion profiles generally show good reproducibility, whereas parameters such as time to peak or incremental area under the curve may exhibit greater day-to-day variation. These findings indicate that CGM captures both stable physiological traits and short-term behavioral or environmental influences, emphasizing the importance of interpreting individual glucose metrics within an appropriate biological context.

Daily variations in sleep, physical activity, meal timing, psychological stress, medication adherence, and preceding dietary intake may all influence glucose regulation independently of the meal itself [29,33,35]. Consequently, a single CGM trace should not be interpreted in isolation but rather as the integrated result of multiple interacting biological and behavioral processes.

### 3.3. Determinants of Glycemic Variability

The considerable variability observed in CGM-derived glucose responses reflects the combined influence of dietary exposures and individual metabolic characteristics [1,2]. Macronutrient composition, food matrix, meal structure, carbohydrate digestibility, and meal timing determine the initial physiological stimulus presented to the body. However, the resulting glycemic phenotype depends equally on the individual’s capacity to digest, absorb, distribute, and metabolize those nutrients [10,20].

Insulin sensitivity, beta-cell function, hepatic glucose regulation [11,25], skeletal muscle glucose uptake [24], adiposity, circadian biology [29], sleep quality, physical activity [33], gut microbiome composition [26], medication use, and other physiological modifiers each influence how nutrients are processed following ingestion. Rather than acting independently, these factors interact continuously to shape the magnitude, duration, and variability of postprandial glucose excursions. The resulting CGM profile therefore represents the cumulative expression of multiple physiological systems rather than the isolated effect of any single nutrient or metabolic pathway.

Recognizing these interacting determinants shifts the interpretation of CGM away from simple descriptions of glucose values toward understanding the biological processes responsible for those observed patterns. This perspective provides the basis for individualized interpretation of glycemic responses and ultimately for precision nutrition.

### 3.4. Why Physiology Matters for Interpreting CGM

Although CGM provides unprecedented insight into dynamic glucose regulation, the physiological meaning of the observed glucose patterns is often less clear than the patterns themselves. Similar glucose excursions may arise through different physiological pathways, while distinct physiological disturbances may produce superficially similar CGM profiles [14,15]. Consequently, interpreting CGM solely through numerical metrics risks overlooking the biological mechanisms that generate those responses [1]. A physiology-based approach recognizes that CGM-derived glycemic phenotypes are integrated manifestations of dietary exposures interacting with individual metabolic characteristics. Rather than representing isolated glucose measurements, these phenotypes reflect the coordinated effects of nutrient digestion and absorption, hormonal regulation, hepatic glucose production, peripheral glucose disposal, and behavioral influences operating within the broader metabolic context [11,13]. Viewed in this way, CGM provides a means of observing the physiological consequences of nutrient metabolism under free-living conditions. This physiology-based perspective forms the conceptual foundation of the framework presented in Figure 1 and Table 1 and establishes the rationale for the following section, which examines the biological processes that generate CGM-derived glycemic phenotypes.

## 4. Physiological Basis of CGM-Derived Glucose Responses

Continuous glucose monitoring provides a dynamic representation of glucose regulation under free-living conditions. However, CGM records the physiological consequences of metabolic regulation rather than the underlying mechanisms themselves [6,15]. Every postprandial glucose profile reflects the integrated effects of nutrient digestion and absorption, hormonal signaling, hepatic glucose production, peripheral glucose disposal, and individual metabolic characteristics [11,13]. Consequently, similar CGM-derived glycemic phenotypes may arise through different physiological pathways, whereas distinct physiological disturbances may produce superficially similar glucose patterns [1,14]. Interpreting CGM therefore requires an understanding of the biological processes that collectively shape postprandial glucose responses.

Glucose homeostasis following meal ingestion is regulated through a coordinated network involving the gastrointestinal tract, pancreas, liver, skeletal muscle, adipose tissue, and endocrine signaling pathways [51]. These systems respond simultaneously to nutrient availability and continuously adapt according to the individual’s metabolic state. Rather than acting independently, they function as an integrated physiological network that determines the magnitude, duration, and recovery of postprandial glucose excursions observed through CGM. Appreciating these interactions provides the mechanistic basis for interpreting glycemic phenotypes and understanding why individuals consuming similar meals often exhibit markedly different glucose responses.

### 4.1. Nutrient Digestion, Absorption, and Gastric Emptying

The postprandial glycemic response begins with the digestion and absorption of dietary nutrients [19,20]. Although carbohydrate quantity remains an important determinant of glucose availability, the rate at which glucose enters the circulation is strongly influenced by food structure, starch digestibility, fiber content, gastric emptying, and interactions among macronutrients within mixed meals [9,12]. These factors collectively determine both the magnitude and temporal profile of glucose appearance following food ingestion.

Gastric emptying serves as one of the principal physiological regulators of postprandial glycemia by controlling the delivery of nutrients to the small intestine [12,52,53]. Meals that slow gastric emptying delay glucose absorption, reduce the rate of glucose appearance in the circulation, and generally produce lower postprandial glucose peaks with more gradual recovery trajectories. Conversely, rapid gastric emptying accelerates intestinal glucose delivery and may contribute to sharp early glucose excursions despite similar carbohydrate exposure. Protein, dietary fat, soluble fiber, and the physical structure of food all influence gastric emptying and therefore modify subsequent glycemic responses [9,10,20].

Beyond gastric emptying, differences in starch structure, food processing, and the surrounding food matrix alter the accessibility of carbohydrates to digestive enzymes. Slowly digestible starches, resistant starch, and minimally processed foods typically prolong glucose absorption compared with rapidly digestible carbohydrates [9,12]. These effects illustrate that carbohydrate quality may be as important as carbohydrate quantity when interpreting postprandial glycemic phenotypes.

Variations in nutrient digestion and absorption contribute to distinct CGM-derived glycemic phenotypes. Sharp early excursions often reflect rapid glucose appearance following accelerated gastric emptying or consumption of highly digestible carbohydrates. In contrast, flatter and more prolonged excursions may result from delayed nutrient delivery, slower carbohydrate digestion, or mixed meal effects rather than improved metabolic regulation alone. Consequently, differences in curve morphology should be interpreted in the context of nutrient absorption kinetics and downstream glucose disposal.

### 4.2. Hormonal Regulation of Postprandial Glucose Responses

The entry of nutrients into the gastrointestinal tract initiates a coordinated hormonal response that regulates postprandial glucose homeostasis [54,55]. Glucose-dependent insulin secretion, glucagon suppression, and incretin mediated amplification of insulin responses collectively influence the efficiency with which glucose is cleared from the circulation. These responses influence not only the magnitude of postprandial glucose excursions but also the rate at which glucose concentrations return toward baseline.

Insulin remains the principal hormone responsible for promoting postprandial glucose disposal [13,56]. Following nutrient ingestion, pancreatic beta-cells increase insulin secretion in proportion to the circulating glucose concentration [57]. Insulin facilitates glucose uptake primarily in skeletal muscle and adipose tissue while simultaneously suppressing hepatic glucose production. In healthy physiology, this coordinated response limits excessive postprandial glucose excursions and promotes efficient glucose recovery. In contrast, impaired early phase insulin secretion or reduced insulin sensitivity delays glucose clearance and contributes to larger or more prolonged postprandial excursions.

The incretin hormones glucagon-like peptide 1 (GLP-1) and glucose-dependent insulinotropic polypeptide (GIP) further modulate this response by enhancing glucose-dependent insulin secretion, suppressing glucagon release, and slowing gastric emptying [54,58,59]. Their activity helps synchronize nutrient absorption with insulin availability, thereby reducing excessive glucose fluctuations following meal ingestion. Although incretin physiology has become an important therapeutic target in type 2 diabetes, endogenous incretin responses also contribute substantially to normal variation in postprandial glucose regulation.

Glucagon plays a complementary role by regulating hepatic glucose output [60,61]. During the postprandial period, appropriate suppression of glucagon limits endogenous glucose production while insulin mediated glucose disposal predominates. In individuals with type 2 diabetes, impaired glucagon suppression may contribute to persistent hepatic glucose release despite nutrient availability, further amplifying postprandial hyperglycemia.

Sharp early excursions with prolonged recovery may reflect impaired first-phase insulin secretion, diminished incretin activity, reduced insulin sensitivity, or inadequate suppression of glucagon. In contrast, more modest excursions accompanied by rapid return to baseline are generally consistent with efficient hormonal regulation of postprandial glucose disposal. Similar glucose peaks may therefore arise through different endocrine mechanisms, emphasizing that interpretation of CGM-derived glycemic phenotypes should consider the broader hormonal context rather than glucose values alone [2,14].

### 4.3. Hepatic Regulation of Postprandial Glucose Homeostasis

The liver occupies a central role in maintaining glucose homeostasis by balancing glucose storage, production, and release according to nutritional status [62,63]. During the postprandial period, hepatic glucose production is normally suppressed while absorbed glucose is preferentially stored as glycogen [61]. This transition depends largely on coordinated insulin signaling and appropriate glucagon suppression.

In type 2 diabetes, hepatic insulin resistance disrupts this regulatory process, allowing endogenous glucose production to persist despite adequate nutrient availability [51]. Consequently, hepatic glucose output may remain elevated during the postprandial period, contributing to sustained hyperglycemia even when dietary glucose absorption has begun to decline. This mechanism is particularly important because postprandial glycemia reflects both exogenous glucose entering the circulation and endogenous glucose released from the liver [5,25].

Persistent hepatic glucose production may also contribute to elevated fasting glucose concentrations, the dawn phenomenon, and prolonged overnight hyperglycemia [61,64]. These observations illustrate that similar postprandial glucose excursions may reflect different relative contributions from dietary glucose absorption and endogenous hepatic glucose release.

Persistent daytime hyperglycemia or an elevated overnight CGM baseline should not automatically be attributed to excessive carbohydrate intake. These glycemic phenotypes may instead reflect impaired suppression of hepatic glucose production resulting from hepatic insulin resistance, inappropriate glucagon signaling, or altered circadian regulation [29]. Consequently, interpretation of these patterns requires consideration of both dietary exposures and endogenous glucose regulation.

### 4.4. Peripheral Glucose Disposal and Metabolic Flexibility

Following nutrient absorption and hormonal regulation, glucose disposal within peripheral tissues becomes the principal determinant of postprandial glucose recovery [62]. Skeletal muscle accounts for the majority of insulin mediated glucose uptake after meal ingestion, while adipose tissue contributes to systemic glucose and lipid homeostasis through coordinated regulation of substrate storage and release [11,24]. The efficiency of these processes depends on both insulin sensitivity and the metabolic flexibility of peripheral tissues [65].

Metabolic flexibility describes the capacity of tissues to appropriately shift between lipid and carbohydrate oxidation according to nutrient availability [65,66]. Metabolic flexibility influences not only the magnitude of postprandial glucose excursions but also the efficiency and consistency of glucose recovery observed through CGM, making it an important determinant of glycemic phenotype. In metabolically healthy individuals, insulin stimulates glucose uptake through coordinated intracellular signaling, promotes glycogen synthesis, and suppresses lipolysis, facilitating efficient clearance of circulating glucose [67,68]. In contrast, insulin resistance impairs these adaptive responses, reducing skeletal muscle glucose uptake while allowing continued release of free fatty acids from adipose tissue. The resulting metabolic environment contributes to prolonged postprandial hyperglycemia despite adequate insulin availability.

Although intracellular pathways involving phosphoinositide 3 kinase, protein kinase B (Akt), AMP-activated protein kinase, mammalian target of rapamycin, and GLUT4 trafficking regulate these processes, their collective physiological consequence is more relevant to CGM interpretation than the individual signaling cascades themselves. Together, these pathways determine the efficiency with which glucose is removed from the circulation following nutrient ingestion [67,68].

Prolonged postprandial excursions with delayed return to baseline may reflect impaired peripheral glucose disposal resulting from reduced insulin sensitivity or diminished metabolic flexibility. In contrast, rapid glucose recovery following comparable dietary exposures generally indicates efficient tissue glucose uptake and coordinated metabolic regulation. Consequently, postprandial recovery trajectories may provide insight into peripheral glucose handling rather than carbohydrate intake alone.

### 4.5. Integration of Physiological Processes in CGM-Derived Glycemic Phenotypes

A central challenge in interpreting CGM-derived glycemic phenotypes is that similar glucose patterns may emerge through different combinations of physiological mechanisms. Conversely, similar physiological disturbances may produce different glycemic phenotypes depending on dietary exposures and individual metabolic characteristics. Postprandial glucose regulation therefore does not arise from any single physiological pathway. Instead, the glycemic response recorded by CGM is consistent with the integrated outcome of nutrient digestion, hormonal regulation, hepatic glucose production, peripheral glucose disposal, and individual metabolic characteristics operating simultaneously [62,63]. These processes continuously interact with behavioral factors such as physical activity, sleep, meal timing, and medication use, producing the dynamic glucose patterns observed under free-living conditions.

A sharp postprandial excursion, for example, may result from rapid carbohydrate absorption, impaired first-phase insulin secretion, reduced skeletal muscle glucose uptake, or a combination of these processes. Likewise, persistent daytime hyperglycemia may reflect continued hepatic glucose production, reduced peripheral glucose disposal, behavioral factors, or medication-related influences. The physiological interpretation of CGM therefore requires consideration of the entire metabolic context rather than any individual mechanism in isolation.

Collectively, nutrient digestion and absorption, hormonal regulation, hepatic glucose control, peripheral glucose disposal, and metabolic flexibility interact to generate the CGM-derived glycemic phenotypes observed under free-living conditions [11,63]. These phenotypes reflect the integrated physiological consequences of dietary exposures interacting with individual metabolic characteristics [1]. Accurate interpretation therefore requires simultaneous consideration of nutrient characteristics, endocrine regulation, hepatic function, peripheral glucose disposal, and the broader metabolic context [14,15]. This physiology-based perspective provides the mechanistic foundation for translating CGM-derived phenotypes into individualized nutritional strategies and establishes the framework for examining how specific dietary exposures shape these phenotypes in the following section.

## 5. Dietary Exposures as Determinants of CGM-Derived Glycemic Phenotypes

Dietary exposures represent the primary physiological stimulus that initiates postprandial glucose regulation [2,16]. However, the glycemic response observed following meal ingestion is determined by substantially more than the absolute quantity of carbohydrate consumed. Macronutrient composition, starch structure, food processing, fiber content, meal structure, and meal timing collectively influence the rate of nutrient absorption, hormonal responses, hepatic glucose regulation, and peripheral glucose disposal established in the preceding section [9,10,69]. Consequently, similar carbohydrate loads may produce different CGM-derived glycemic phenotypes depending on how nutrients are delivered, absorbed, and metabolized.

Viewing dietary exposures through this physiological perspective shifts the interpretation of CGM beyond nutrient counting alone. Rather than representing isolated effects of carbohydrates, proteins, or fats, postprandial glucose profiles reflect the integrated biological response to the complete dietary context. This section therefore examines how specific dietary characteristics modify physiological processes and how those modifications become observable as distinct CGM-derived glycemic phenotypes.

### 5.1. Dietary Exposures

Dietary carbohydrate remains the principal determinant of postprandial glucose appearance, yet increasing evidence demonstrates that carbohydrate quantity alone incompletely explains the variability observed in CGM-derived glycemic responses [19,20]. The structural characteristics of carbohydrate containing foods, including starch digestibility, food processing, fiber content, and the surrounding food matrix, substantially influence the rate of glucose absorption and the subsequent physiological response.

Carbohydrate quantity remains an important determinant of postprandial glucose exposure, with larger carbohydrate loads generally producing greater glucose excursions and longer periods above range. However, the relationship between carbohydrate quantity and glycemic response is not strictly linear because nutrient absorption, hormonal regulation, hepatic glucose regulation, peripheral glucose disposal, and individual metabolic characteristics all modify the physiological consequences of a given carbohydrate exposure. Consequently, carbohydrate quantity provides only a partial explanation for the variability observed in CGM-derived glycemic phenotypes.

Starch functionality represents one of the most important determinants of postprandial glucose kinetics. Highly digestible starches undergo rapid enzymatic hydrolysis, resulting in accelerated intestinal glucose absorption and earlier postprandial glucose peaks. In contrast, slowly digestible starches and resistant starch delay glucose appearance within the circulation, producing flatter glucose excursions and more gradual recovery trajectories. Controlled feeding studies indicate that starch digestibility, food structure, particle size, and processing can meaningfully alter postprandial glucose responses even when carbohydrate content is comparable [70,71].

The surrounding food matrix further modifies carbohydrate availability. Whole grains, legumes, minimally processed foods, and fiber-rich dietary patterns slow nutrient absorption through alterations in gastric emptying, intestinal viscosity, and physical accessibility of carbohydrates to digestive enzymes [12]. Conversely, extensive food processing often increases starch accessibility and accelerates glucose appearance despite similar macronutrient composition. These observations illustrate that foods containing comparable carbohydrate quantities may generate substantially different glycemic phenotypes because of differences in nutrient structure rather than nutrient content alone.

Dietary fiber contributes additional physiological effects beyond slowing glucose absorption [72]. Soluble fibers increase intestinal viscosity, delay gastric emptying, and attenuate postprandial glucose excursions, while fermentable fibers undergo microbial metabolism to produce short chain fatty acids that influence glucose regulation through multiple endocrine and metabolic pathways. Although these downstream mechanisms remain an active area of investigation, the cumulative physiological consequence is generally reflected by reduced postprandial glucose variability and improved glycemic stability.

Variations in carbohydrate quality, starch functionality, food processing, and fiber content contribute to distinct CGM-derived glycemic phenotypes. Sharp early excursions and rapid glucose peaks commonly reflect highly digestible carbohydrate sources and accelerated glucose appearance, whereas flatter and more prolonged excursions may result from slower digestion, increased fiber content, or food matrix effects rather than improved metabolic regulation alone. Consequently, similar carbohydrate quantities should not be assumed to produce equivalent glycemic responses without considering the physiological determinants of nutrient absorption.

### 5.2. Meal Structure

Although individual macronutrients influence postprandial glucose regulation, they are rarely consumed in isolation. Most meals contain varying combinations of carbohydrates, proteins, fats, and fiber that interact throughout digestion, absorption, and metabolism [10,21]. Consequently, the physiological response to a mixed meal often differs substantially from that predicted by the carbohydrate content alone. Meal structure therefore represents an important determinant of CGM-derived glycemic phenotypes.

Protein influences postprandial glucose regulation through several complementary mechanisms, with evidence derived from both studies in adults with type 2 diabetes and foundational mechanistic investigations in other populations, including individuals with type 1 diabetes [73,74,75,76]. In addition to stimulating glucose-dependent insulin secretion, protein promotes incretin release and may modestly delay gastric emptying when consumed as part of mixed meals. These responses frequently attenuate early glucose excursions without necessarily altering total carbohydrate exposure. Recent intervention studies have further reported that protein preloads administered before carbohydrate containing meals may reduce postprandial glucose excursions, although the magnitude of this effect appears to depend on the individual’s underlying metabolic phenotype and degree of insulin resistance [9,77].

Dietary fat also modifies postprandial glucose regulation by slowing gastric emptying and altering nutrient absorption kinetics [69,78,79]. While this often delays the appearance of glucose in the circulation, high fat meals may prolong postprandial hyperglycemia through delayed nutrient delivery and sustained substrate availability. Consequently, meals with similar carbohydrate content but different fat composition may produce markedly different glucose trajectories despite comparable total glucose exposure. Evidence from mixed-meal studies, including investigations in individuals with type 1 diabetes, supports these physiological effects, although direct evidence in adults with type 2 diabetes remains more limited [80,81].

These interactions become particularly evident in mixed meals, where nutrient sequencing and food combinations influence multiple physiological pathways simultaneously. Consuming vegetables, protein, or fiber before carbohydrates has consistently been associated with smaller postprandial glucose excursions in many controlled feeding studies, illustrating that the order in which nutrients are consumed may influence glycemic responses independently of total carbohydrate intake [21].

Meal structure contributes substantially to CGM-derived glycemic phenotypes. Reduced excursion amplitude following comparable carbohydrate loads is often indicative of altered nutrient delivery, delayed gastric emptying, or enhanced hormonal responses rather than reduced carbohydrate exposure alone. Likewise, prolonged postprandial excursions may result from delayed nutrient absorption associated with higher fat content rather than impaired glucose regulation. Consequently, meal composition and nutrient sequencing should be considered when interpreting postprandial CGM responses.

### 5.3. Meal Timing

The physiological response to food is influenced not only by what is consumed, but also by when it is consumed [27,30,31]. Circadian variation in insulin sensitivity, hepatic glucose production, gastrointestinal physiology, and hormonal regulation results in predictable changes in metabolic efficiency throughout the day. Consequently, identical meals consumed at different times may produce substantially different CGM-derived glycemic phenotypes.

Several intervention studies have shown that earlier carbohydrate consumption is generally associated with lower postprandial glucose excursions than equivalent meals consumed later in the day [23,27]. Conversely, late evening meals frequently produce prolonged postprandial hyperglycemia and elevated overnight glucose concentrations, reflecting reduced insulin sensitivity and altered hepatic glucose regulation during the biological evening [28]. Time-restricted eating and other chrononutrition strategies similarly appear to influence glycemic control, although their effects likely depend on individual metabolic characteristics, medication use, and habitual eating patterns [30,32].

Meal timing also interacts with physical activity and sleep. Postprandial exercise, particularly when performed within the first hour after meal ingestion, may attenuate glucose excursions by increasing peripheral glucose uptake independently of insulin action [82,83]. Likewise, irregular meal timing or inconsistent eating schedules may contribute to greater glycemic variability by disrupting the coordination between nutrient delivery and endogenous circadian regulation [27].

Meal timing contributes to distinct CGM-derived glycemic phenotypes. Elevated evening or overnight glucose profiles may reflect circadian reductions in metabolic efficiency rather than differences in meal composition alone. Similarly, attenuated postprandial excursions following exercise or earlier carbohydrate consumption often reflect improved physiological glucose disposal rather than reduced dietary exposure. Interpretation of CGM-derived phenotypes should therefore consider both the timing of nutrient intake and the temporal context in which glucose responses occur.

Collectively, dietary exposures shape CGM-derived glycemic phenotypes through coordinated effects on nutrient absorption, hormonal regulation, hepatic glucose control, peripheral glucose disposal, and metabolic flexibility rather than through isolated nutrient effects. However, dietary exposures represent only one component of the physiological response. The magnitude, timing, and recovery of the resulting glycemic phenotype remain strongly influenced by the individual’s metabolic characteristics, which determine how nutrients are processed following ingestion. This interaction between dietary exposures and individual physiology provides the foundation for the following section, which examines the intrinsic biological factors that modify CGM responses.

## 6. Individual Metabolic Characteristics Modifying CGM Responses

The physiological response to food is determined not only by the characteristics of the meal itself but also by the metabolic characteristics of the individual consuming it. While dietary exposures initiate postprandial glucose regulation, the resulting glycemic phenotype reflects the capacity of multiple physiological systems to process, distribute, and utilize absorbed nutrients. Consequently, individuals consuming identical meals frequently exhibit different CGM-derived glucose responses because their underlying metabolic physiology differs [1,2].

This interindividual heterogeneity extends beyond traditional clinical measures such as body weight, HbA1c, or diabetes duration. Insulin sensitivity, beta-cell function, body composition, gut microbial activity, habitual physical activity, sleep, circadian regulation, medication use, and emerging molecular characteristics each influence the physiological response to nutrient exposure [13,27,62,82,84]. Rather than acting independently, these factors interact continuously to modify digestion, hormonal regulation, hepatic glucose production, and peripheral glucose disposal, thereby shaping the glycemic phenotypes observed through CGM [63]. Understanding these modifiers is therefore essential for interpreting CGM-derived glucose patterns and translating them into individualized nutritional strategies.

### 6.1. Insulin Sensitivity and Beta-Cell Function

Among the many factors contributing to interindividual variability, insulin sensitivity and beta-cell function remain the principal physiological determinants of postprandial glucose regulation [13,62,85]. Together, they determine both the efficiency of glucose disposal and the capacity to mount an appropriate insulin response following nutrient ingestion. Individuals with similar dietary exposures may therefore exhibit markedly different glycemic phenotypes because of differences in these underlying metabolic characteristics.

Reduced insulin sensitivity limits glucose uptake by peripheral tissues while diminishing insulin mediated suppression of hepatic glucose production [86]. As insulin resistance progresses, beta-cells initially compensate through increased insulin secretion, but this adaptive response gradually becomes insufficient as beta-cell function declines [87]. The resulting combination of impaired insulin action and reduced secretory capacity contributes to larger postprandial excursions, delayed glucose recovery, and greater glycemic variability.

Recent studies using CGM have further reported that individuals with similar HbA1c values may display distinct glycemic phenotypes reflecting different combinations of insulin resistance and beta-cell dysfunction [7,14]. These observations suggest that CGM patterns provide insight into underlying metabolic heterogeneity beyond conventional measures of long-term glycemic control.

Insulin sensitivity and beta-cell function contribute to distinct CGM-derived glycemic phenotypes. Sharp early excursions with prolonged recovery frequently reflect impaired early insulin secretion, whereas sustained postprandial hyperglycemia or persistent daytime elevations may indicate reduced peripheral insulin sensitivity, impaired hepatic glucose regulation, or a combination of both. Consequently, similar dietary exposures may generate different glucose responses because of differences in underlying metabolic physiology rather than food intake alone.

### 6.2. Body Composition and Obesity

Body composition influences postprandial glucose regulation independently of total body weight. Although obesity is commonly associated with insulin resistance, individuals with similar body mass index may differ substantially in visceral adiposity, skeletal muscle mass, hepatic fat accumulation, and overall metabolic health [62,63,86]. These differences contribute to considerable variability in glucose handling following identical dietary exposures.

Visceral adiposity promotes chronic low-grade inflammation, ectopic lipid deposition, and impaired insulin signaling, thereby reducing glucose uptake by peripheral tissues while increasing hepatic glucose production [88,89]. In contrast, preservation of skeletal muscle mass enhances insulin mediated glucose disposal and improves metabolic flexibility during the postprandial period [90]. Recent studies suggest that body composition, including visceral adiposity and skeletal muscle mass, may provide greater insight into glycemic responses than body weight or body mass index alone, highlighting the importance of distinguishing metabolic health from body size [47].

Body composition contributes to distinct CGM-derived glycemic phenotypes. Persistent daytime hyperglycemia, prolonged postprandial recovery, and greater glycemic variability may reflect impaired glucose disposal associated with visceral adiposity or reduced skeletal muscle mass rather than differences in dietary intake alone. Consequently, interpretation of CGM-derived phenotypes should consider body composition alongside conventional clinical measures.

### 6.3. Physical Activity

Physical activity modifies postprandial glucose regulation through both insulin-dependent and insulin-independent mechanisms. Muscle contraction stimulates glucose uptake independently of insulin, while habitual exercise improves insulin sensitivity, mitochondrial function, and metabolic flexibility [65,82,83]. These adaptations influence not only the magnitude of postprandial glucose excursions but also the consistency of glucose regulation over time.

Acute postprandial exercise has emerged as an important modifier of CGM responses. Recent studies demonstrate that walking or moderate intensity exercise performed shortly after meal ingestion attenuates glucose excursions and reduces time spent above range [65,91,92]. The magnitude of this benefit appears to depend on exercise timing, meal composition, and the individual’s underlying metabolic characteristics, suggesting that physical activity modifies the physiological response to food rather than acting independently of it.

Physical activity contributes to glycemic phenotypes characterized by reduced postprandial excursion amplitude, faster glucose recovery, and lower overall glycemic variability [91,93]. Conversely, the absence of these responses following comparable exercise may indicate impaired metabolic flexibility or more advanced insulin resistance. Interpretation of meal-related CGM responses should therefore consider both habitual and acute physical activity.

### 6.4. Sleep

Sleep is increasingly recognized as an important regulator of glucose homeostasis [27,35]. Experimental sleep restriction and chronic sleep disruption impair insulin sensitivity, alter autonomic regulation, increase inflammatory signaling, and reduce beta-cell responsiveness, collectively diminishing the efficiency of postprandial glucose regulation.

CGM studies demonstrate that poor sleep quality and irregular sleep duration are associated with higher mean glucose concentrations, greater glycemic variability, and less predictable postprandial responses [3,94,95]. These relationships appear to persist even after accounting for dietary intake, emphasizing that glucose regulation should prompt consideration of broader physiological state rather than nutritional exposure alone.

Sleep-related disturbances contribute to glycemic phenotypes characterized by increased day-to-day variability, elevated overnight glucose, and less efficient postprandial recovery [35,94]. These patterns should be interpreted within the context of recent sleep behavior rather than attributed solely to dietary factors.

Because sleep and circadian regulation influence glucose metabolism through complementary but distinct physiological pathways [27,28], alterations in sleep continuity are frequently accompanied by changes in the temporal organization of glucose regulation. The influence of endogenous circadian rhythms on CGM-derived glycemic phenotypes is discussed in the following section.

### 6.5. Circadian Regulation

Glucose metabolism follows a circadian rhythm governed by central and peripheral molecular clocks that coordinate insulin sensitivity, hepatic glucose production, gastrointestinal physiology, and hormonal secretion throughout the day [27,28]. As a result, metabolic responses to identical meals vary according to the timing of nutrient intake.

Several recent intervention studies have reported that carbohydrate consumption later in the day frequently produces larger and more prolonged postprandial excursions than comparable morning meals [96,97]. Similarly, late evening eating and circadian misalignment have been associated with elevated overnight glucose concentrations and reduced time in range [98,99]. These findings suggest that temporal organization represents an integral component of metabolic physiology rather than simply a behavioral habit.

Circadian regulation contributes to glycemic phenotypes characterized by elevated overnight glucose, evening hyperglycemia, and exaggerated responses to identical meals consumed later in the day [98,100]. These patterns may reflect endogenous circadian variation in metabolic efficiency rather than differences in dietary composition alone.

### 6.6. Medications

Glucose-lowering medications modify nearly every aspect of postprandial glucose regulation and therefore represent an important consideration when interpreting CGM-derived phenotypes [101]. Agents such as metformin, GLP-1 receptor agonists, SGLT2 inhibitors, insulin, and sulfonylureas influence glucose metabolism through distinct physiological mechanisms that may substantially alter CGM responses independent of dietary exposures [58,59,63].

Consequently, similar CGM profiles may reflect different underlying physiology depending on the therapeutic context. Likewise, improvements in glycemic variability following dietary interventions may be partially attributable to concurrent pharmacological effects unless medication regimens remain stable [6,102].

Medication use modifies glycemic phenotypes through mechanisms that may mimic or mask physiological responses to dietary interventions. Interpretation of CGM-derived glucose patterns should therefore consider both the nutritional intervention and the background pharmacological environment.

### 6.7. Gut Microbiome

The gut microbiome has emerged as a potential contributor to interindividual variability in postprandial glucose regulation [1,2]. Microbial metabolism influences glucose homeostasis through production of short chain fatty acids, bile acid metabolism, intestinal barrier function, and interactions with enteroendocrine signaling pathways [103]. These mechanisms provide plausible biological links between microbial composition and glycemic regulation [104,105].

Although several studies have identified associations between microbial signatures and individualized glycemic responses, reproducibility across populations remains inconsistent [1,2]. Differences in diet, geography, analytical methodology, and host characteristics continue to limit direct clinical translation. Although concepts such as enterotypes, postbiotics, and precision microbiome-guided nutrition have generated considerable interest, current evidence remains insufficient to support their routine clinical application. Nevertheless, accumulating evidence suggests that the gut microbiome may contribute to the metabolic heterogeneity observed among individuals consuming similar diets [2,106].

Gut microbial composition may contribute to glycemic phenotypes characterized by differential responses to similar dietary exposures. However, current evidence remains insufficient to support routine microbiome-based interpretation of CGM responses, and these findings should be considered emerging rather than definitive.

### 6.8. Emerging Omics and Molecular Phenotyping

Advances in genomics, nutrigenomics, nutrigenetics, metabolomics, proteomics, and machine learning are expanding the ability to characterize metabolic heterogeneity beyond traditional clinical measures [107,108,109]. These approaches seek to identify biological signatures that explain why individuals with similar clinical characteristics often display markedly different glycemic responses, building upon foundational observations of interindividual metabolic variability [1,2].

Recent investigations integrating CGM with multi-omic profiling suggest that individualized glucose responses may eventually be interpreted using combinations of physiological, molecular, behavioral, and environmental information. Although these approaches remain largely investigational, they illustrate a shift from population-based nutritional recommendations toward increasingly individualized metabolic characterization.

Emerging multi-omic approaches may ultimately refine the interpretation of CGM-derived glycemic phenotypes by identifying biological mechanisms that are not apparent from glucose data alone [110]. At present, however, these technologies should be viewed as promising research tools that require further validation before widespread clinical implementation [106].

Collectively, individual metabolic characteristics influence every stage of postprandial glucose regulation, from nutrient absorption and hormonal signaling to hepatic glucose production and peripheral glucose disposal [62,63]. These interacting factors explain much of the variability in CGM-derived glycemic phenotypes observed following similar dietary exposures. Building on this physiological foundation, the following section presents a framework for interpreting these phenotypes and translating them into individualized nutritional strategies.

## 7. Interpreting CGM-Derived Glycemic Phenotypes Through a Physiological Framework

### 7.1. From Glucose Metrics to Glycemic Phenotypes

Continuous glucose monitoring generates a broad range of quantitative metrics that characterize glucose behavior over time, including mean glucose, postprandial excursions, time in range, time above range, time below range, coefficient of variation, mean amplitude of glycemic excursions, peak glucose, time to peak, and incremental area under the curve [15,101]. These measurements provide valuable information regarding glycemic control and variability, yet they primarily describe what is observed rather than why it occurs [6].

Glycemic phenotypes extend beyond individual glucose metrics by providing a physiological interpretation of observed glucose patterns. Rather than representing diagnoses, glycemic phenotypes describe recurring patterns of glucose regulation that emerge from the interaction between dietary exposures, underlying metabolic physiology, and individual metabolic characteristics. A sharp early excursion, prolonged postprandial excursion, persistent daytime hyperglycemia, elevated overnight baseline, or high day-to-day variability each represents an observable glucose pattern that may arise through multiple physiological pathways [14].

The distinction between glucose metrics and glycemic phenotypes is central to interpreting CGM. Glucose metrics quantify what is observed, whereas glycemic phenotypes seek to explain what those observations may reflect biologically. Mechanistic interpretation therefore does not replace quantitative CGM metrics, but builds upon them by integrating dietary context, metabolic physiology, and individual variability into a coherent physiology-based framework. Accordingly, glycemic phenotypes may be viewed as physiologically interpreted CGM patterns rather than quantitative glucose metrics alone.

**Figure 2:**
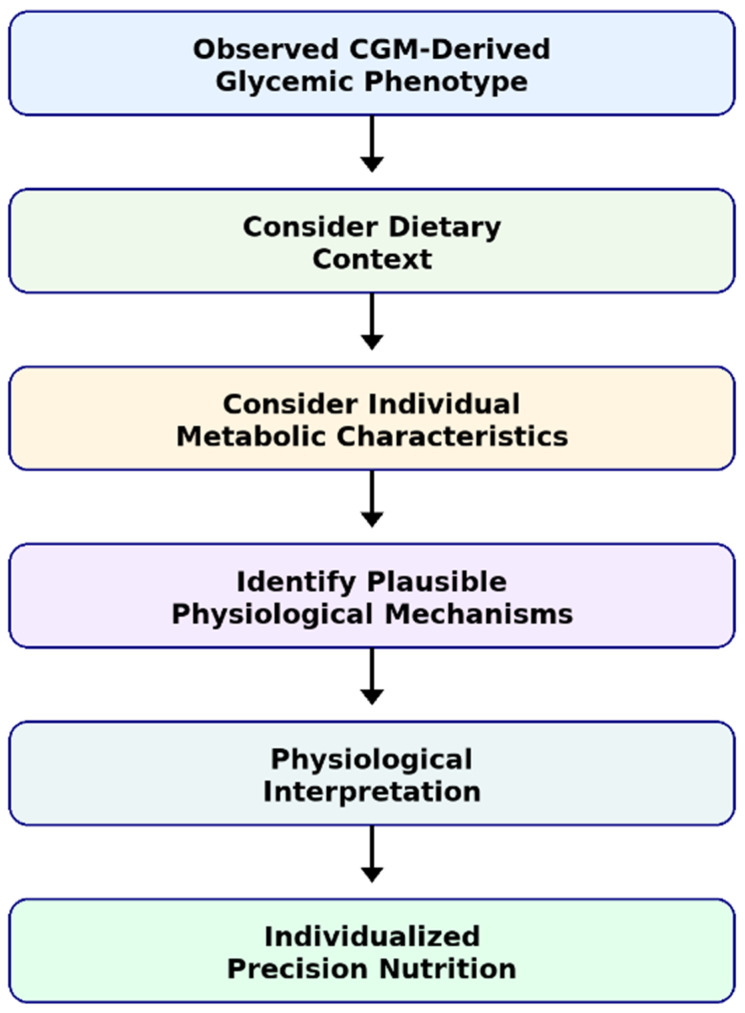
Physiological framework for interpreting CGM-derived glycemic phenotypes and informing precision nutrition. This figure presents a conceptual, non-diagnostic framework. Arrows indicate hypothesized or integrated relationships and do not imply one-to-one causal inference from CGM patterns. Created in BioRender. Belani, Jitendra D. (2026) (https://app.biorender.com/illustrations/canvas-beta/6a733e6ac1d6725a443b84dc, accessed on 8 May 2026).

The objective of physiological interpretation is not to infer a single causal mechanism from an individual glucose trace, but rather to identify plausible physiological processes that may explain the observed glycemic phenotype within its dietary, behavioral, pharmacological, and clinical context. Similar glycemic phenotypes may therefore arise through different physiological pathways, while similar physiological disturbances may produce different phenotypes depending on the surrounding metabolic environment. The physiological interpretation process outlined within the conceptual framework (Figure 1) and summarized in Table 1 provides a structured approach for linking observed CGM-derived glycemic phenotypes to plausible biological mechanisms. The phenotypes proposed here are conceptual physiological categories intended to facilitate interpretation and should not be viewed as formally validated classifications. Figure 2 illustrates the proposed interpretive workflow through which these phenotypes are evaluated within their dietary, physiological, behavioral, and clinical context.

### 7.2. Interpreting Common CGM-Derived Glycemic Phenotypes

#### 7.2.1. Sharp Early Excursion

A sharp early excursion is characterized by rapid postprandial glucose elevation shortly after meal ingestion. This phenotype may reflect rapid carbohydrate absorption associated with highly digestible starches, reduced dietary fiber, accelerated gastric emptying, impaired first-phase insulin secretion, diminished incretin activity, reduced peripheral glucose disposal, or combinations of these mechanisms [48,54,63,82].

A sharp early excursion should not be interpreted as evidence of excessive carbohydrate intake in isolation. Rather, it represents a glycemic phenotype that may arise through multiple physiological mechanisms, emphasizing the importance of integrating dietary composition, hormonal regulation, and individual metabolic characteristics when interpreting CGM responses. Recognition of this phenotype may help generate hypotheses regarding the physiological contributors to the observed response and guide consideration of individualized nutritional strategies.

#### 7.2.2. Prolonged Postprandial Excursion

A prolonged postprandial excursion is characterized by delayed return of glucose concentrations toward baseline following a meal. This phenotype may reflect delayed glucose clearance resulting from peripheral insulin resistance, reduced metabolic flexibility, diminished insulin sensitivity, delayed gastric emptying associated with mixed meals, or combinations of these physiological mechanisms [62,65,69].

Unlike a sharp early excursion, which often reflects the rate of glucose appearance, a prolonged postprandial excursion reflects delayed recovery after a specific meal. The persistence of elevated glucose concentrations suggests impaired glucose disposal rather than accelerated nutrient absorption alone.

This phenotype may help identify physiological processes that warrant consideration when developing individualized nutritional approaches aimed at improving glucose disposal and postprandial recovery.

#### 7.2.3. Persistent Daytime Hyperglycemia

Persistent daytime hyperglycemia is characterized by sustained elevation of glucose concentrations across multiple meals and fasting intervals [13,63,86]. This phenotype may arise through impaired hepatic insulin sensitivity, inadequate suppression of hepatic glucose production, progressive beta cell dysfunction, reduced skeletal muscle glucose uptake, medication-related influences, or combinations of these mechanisms.

In contrast to a prolonged postprandial excursion, persistent daytime hyperglycemia is commonly associated with sustained glucose elevation throughout the day rather than delayed recovery from a single meal. This phenotype may be consistent with broader disturbances in overall glucose regulation involving endogenous glucose production, peripheral glucose disposal, medication-related effects, behavioral factors, or combinations of these influences.

Persistent daytime hyperglycemia should be interpreted within the context of dietary exposures together with overall metabolic function. Nutritional interventions alone may be insufficient when hepatic or peripheral metabolic dysfunction predominates.

#### 7.2.4. Elevated Overnight Baseline

An elevated overnight baseline is characterized by sustained nocturnal glucose concentrations despite the absence of nutrient intake. This phenotype may reflect increased nocturnal hepatic glucose production, the dawn phenomenon, circadian dysregulation, late evening eating, sleep disruption, medication effects, or combinations of these physiological contributors [27,28,98,100].

Because this phenotype develops during the fasting state, it may reflect endogenous physiological regulation rather than the direct glycemic effect of a recent meal. Interpretation therefore requires consideration of hepatic glucose regulation, circadian biology, sleep, and medication use.

Elevated overnight glucose may support consideration of strategies addressing meal timing, circadian alignment, and overall metabolic regulation rather than focusing exclusively on evening carbohydrate intake.

#### 7.2.5. High Day-to-Day Variability

High day-to-day variability is commonly associated with inconsistent glycemic responses across similar dietary exposures. This phenotype may arise from irregular meal timing, inconsistent carbohydrate intake, fluctuations in physical activity, sleep disruption, medication adherence, reduced metabolic flexibility, or combinations of behavioral and physiological factors [2,50,111].

Unlike postprandial phenotypes that recur consistently under comparable conditions, high day-to-day variability may reflect instability of the broader physiological environment rather than any single dietary exposure. Consequently, interpretation should first consider whether the observed variability represents reproducible biological behavior or transient fluctuations resulting from changes in lifestyle, medication use, illness, psychological stress, or other contextual factors. Reduced metabolic flexibility may further amplify these effects by diminishing the body’s ability to maintain consistent glucose regulation in response to everyday physiological challenges [65].

Improving the consistency of dietary and lifestyle behaviors may therefore be as important as modifying nutrient composition when developing individualized nutritional strategies. Repeated CGM assessment under comparable conditions may also help distinguish persistent physiological patterns from normal day-to-day variation, thereby improving confidence in phenotype-guided interpretation.

### 7.3. Strengths and Limitations of Physiological Interpretation

A physiology-based interpretation of CGM provides a broader understanding of glucose regulation than isolated glucose metrics because it integrates dietary exposures, metabolic physiology, and individual clinical context. Nevertheless, glycemic phenotypes should not be interpreted deterministically. Similar glucose patterns may arise through different physiological mechanisms, while similar physiological disturbances may produce different glycemic phenotypes depending on dietary composition, medication use, behavioral factors, and the surrounding metabolic environment [1,2].

Several additional limitations warrant consideration. CGM measures interstitial glucose rather than organ specific physiology and therefore represents the integrated consequence of multiple biological processes rather than direct measurement of any single mechanism [15,101]. Medication effects, variations in sleep and physical activity, psychological stress, illness, and inconsistencies in dietary assessment may each influence observed glucose patterns [106]. Furthermore, some CGM metrics demonstrate greater reproducibility than others, emphasizing that physiological interpretation should consider the stability of the observed phenotype in addition to its absolute magnitude [50].

Accordingly, physiological interpretation should be viewed as a framework for identifying plausible biological explanations rather than inferring definitive mechanistic diagnoses from individual glucose traces. Integration of CGM with dietary assessment, clinical characteristics, and emerging physiological biomarkers may provide more robust interpretation than reliance on CGM alone [109,110].

### 7.4. Integrating Physiology into Precision Nutrition

The framework proposed in this review shifts precision nutrition from a predominantly algorithm driven concept toward a physiology informed interpretive process. Rather than assuming that glucose data alone determine dietary recommendations, this approach recognizes that observed glycemic phenotypes require physiological interpretation before meaningful nutritional decisions can be made.

Within this framework, CGM serves as a physiological lens through which dietary exposures, metabolic regulation, and individual metabolic characteristics can be integrated. Nutritional strategies are therefore informed not simply by observed glucose values, but by the plausible physiological processes contributing to those patterns. Precision nutrition thus becomes a physiology-informed interpretive process rather than the application of predetermined dietary algorithms.

Emerging technologies, including machine learning, metabolomics, microbiome profiling, and digital health platforms, may further strengthen this interpretive approach by integrating multiple biological and behavioral data streams [94,106,109,110]. However, these technologies should complement physiological interpretation rather than replace it. Their greatest potential lies in refining understanding of metabolic heterogeneity and supporting individualized decision-making within an established physiological framework [2,110].

Successful clinical implementation will require more than technological innovation. Clinicians will need practical interpretation frameworks, appropriate training, and decision support tools capable of integrating dietary information with physiological context. Equally important, patients must remain active participants in the interpretive process, as meaningful precision nutrition depends on sustained engagement, individualized counseling, and shared clinical decision-making rather than data generation alone.

CGM-derived glycemic phenotypes represent the integrated physiological consequences of dietary exposures interacting with individual metabolic characteristics. Interpreting these phenotypes through a physiology-based framework provides a structured approach for linking observed glucose patterns with plausible biological mechanisms. This framework provides the conceptual bridge between quantitative glucose monitoring and individualized precision nutrition while acknowledging the inherent complexity and heterogeneity of glucose regulation in type 2 diabetes.

## 8. Translating Physiological Interpretation into Precision Nutrition

The physiological framework developed in the preceding sections provides a basis for translating CGM observations into individualized nutritional care. CGM alone does not generate precision nutrition recommendations. Its clinical value depends on whether observed glucose patterns can be evaluated in relation to dietary context, individual metabolic characteristics, and plausible physiological mechanisms. Translation therefore requires progression from glucose measurement to phenotype recognition, physiology-based assessment, and individualized action.

The interpretive workflow proposed in Figure 2 illustrates how observed CGM-derived glycemic phenotypes can inform precision nutrition through the sequential consideration of dietary context, individual metabolic characteristics, and plausible physiological mechanisms. This process preserves the value of established dietary guidance while allowing its application to be adapted to the physiological and clinical context of the individual [106,112].

### 8.1. From Physiological Interpretation to Individualization

Population-based nutritional recommendations remain central to the management of type 2 diabetes [16,113]. Dietary patterns emphasizing nutrient quality, appropriate energy intake, fiber-rich foods, minimally processed carbohydrate sources, and cardiometabolic risk reduction provide an essential foundation for care. However, population averages cannot fully represent the heterogeneity of glycemic responses observed among individuals consuming similar foods or following comparable dietary patterns [1,2].

Individualization begins by determining how broadly applicable recommendations interact with the metabolic characteristics of a particular person [47]. Within the proposed framework, an observed CGM pattern is first evaluated as a glycemic phenotype rather than treated as an isolated glucose value. Dietary composition, meal structure, meal timing, physical activity, sleep, medication use, insulin sensitivity, beta-cell function, and other relevant factors are then considered to identify plausible contributors to that phenotype. This mechanistic assessment may help identify which aspects of nutrition warrant further evaluation or consideration.

This approach does not imply that every glucose excursion requires intervention. Nor does it assume that a single meal response provides sufficient evidence for a lasting dietary recommendation. Individualization requires attention to the reproducibility of the phenotype, its clinical significance, the circumstances under which it occurs, and whether the proposed strategy supports overall nutritional quality and long-term metabolic health [50]. CGM-informed nutrition should therefore be based on recurring patterns interpreted within a broader clinical context rather than isolated responses to individual foods.

### 8.2. Precision Nutrition as an Interpretive Process

Precision nutrition is frequently described as the use of biological, behavioral, and environmental data to predict individual responses to food [2,106]. Although precision nutrition has advanced substantially in recent years, most available studies remain short in duration, use heterogeneous dietary interventions and CGM protocols, and provide limited evidence regarding long-term clinical outcomes. Current evidence suggests that precision nutrition may improve selected short-term metabolic outcomes, particularly postprandial glycemia; however, evidence supporting sustained long-term clinical benefits remains limited and heterogeneous [114]. Prediction is an important component of this process, but prediction alone does not explain why a particular response occurs or whether the same recommendation will remain appropriate across changing physiological and behavioral conditions. A physiology-informed approach adds a mechanistic assessment between data collection and nutritional action.

Emerging intervention studies provide preliminary clinical support for CGM-informed and personalized nutritional approaches in adults with type 2 diabetes, although important limitations remain. A randomized crossover pilot trial demonstrated that a personalized postprandial-targeting diet guided by predicted glycemic responses improved several CGM-derived glycemic measures compared with a Mediterranean-style diet in adults with newly diagnosed type 2 diabetes; however, the small sample size and subsequent uncontrolled extension limit the certainty and generalizability of these findings [115]. Similarly, randomized studies using CGM to support patient-driven lifestyle modification have reported improvements in glycemic control and self-management, suggesting that CGM-informed behavioral interventions may facilitate dietary change in routine clinical practice [116]. The UNITE trial likewise reported improvements in several glycemic measures following nutrition-focused CGM education; however, changes in time in range were not significantly different from those achieved with a self-directed approach, illustrating both the potential and current limitations of structured CGM-guided nutrition interventions [102]. More broadly, a recent systematic review and meta-analysis concluded that CGM-guided lifestyle interventions may improve selected glycemic outcomes in adults with type 2 diabetes. Nevertheless, substantial heterogeneity in intervention design, patient populations, and outcome measures limits firm conclusions regarding long-term clinical benefit [117]. Together, these studies support the clinical potential of CGM-informed nutritional personalization while underscoring the need for physiology-informed interpretation and larger prospective trials.

Within this framework, precision nutrition does not proceed directly from CGM data to an algorithmic dietary recommendation. Rather, it begins with recognition of a CGM-derived glycemic phenotype, followed by evaluation of the dietary and clinical context, consideration of plausible physiological mechanisms, and development of an individualized nutritional strategy. The glycemic phenotypes summarized in Table 1 provide illustrative examples of how observed CGM patterns may be linked to plausible biological mechanisms that can inform individualized nutritional assessment.

This distinction is fundamental. Precision nutrition becomes an interpretive process rather than the application of predetermined dietary algorithms. The same glucose pattern may lead to different considerations depending on the mechanism that is most likely contributing to it. For example, a sharp early excursion associated with rapid carbohydrate absorption may warrant a different nutritional focus from a similar excursion occurring in the context of impaired early insulin secretion. Likewise, persistent daytime hyperglycemia related to broader metabolic dysfunction should not be approached as though it were solely the consequence of one food or meal.

This interpretive approach also allows recommendations to remain responsive over time. Glucose regulation may change with weight loss, medication adjustment, physical activity, sleep, disease progression, or improvement in metabolic health [2,4]. Precision nutrition should therefore be viewed as an iterative process in which dietary strategies are implemented, observed through repeated CGM assessment, interpreted within the updated physiological context, and refined when necessary.

### 8.3. Emerging Technologies Supporting Physiology-Informed Interpretation

**Computational approaches.** Artificial intelligence and machine learning may increase the capacity to analyze the volume and complexity of data generated by CGM and related digital health technologies [40,109,110,118]. These methods can identify recurring glucose patterns, examine responses across multiple meals, recognize interactions among dietary and behavioral variables, and generate predictions that may not be apparent through manual review. When combined with dietary records, activity data, sleep measures, medication information, and clinical characteristics, computational models may support more comprehensive characterization of individual glycemic phenotypes.

Predictive accuracy, however, should not be equated with mechanistic understanding. An algorithm may identify that a person is likely to experience a large glucose excursion after a particular meal without clarifying whether the response reflects carbohydrate absorption kinetics, impaired insulin secretion, reduced peripheral disposal, meal timing, prior activity, sleep disruption, or another contributing factor. Models designed primarily to predict glucose values may therefore reproduce associations without establishing biologically meaningful explanations.

The most useful role of artificial intelligence within the proposed framework is to support mechanistic interpretation rather than bypass clinical reasoning. Computational tools may help distinguish reproducible patterns from random variation, identify contextual factors associated with specific responses, and generate hypotheses regarding the physiological mechanisms most consistent with the available data [94]. Their outputs would then inform clinician and patient decision-making rather than function as autonomous dietary prescriptions.

Digital twins represent a further extension of computational modeling in which a dynamic representation of an individual is updated using longitudinal clinical, behavioral, and sensor-derived data. In principle, such models could simulate responses to alternative meal compositions, timing patterns, activity levels, or treatment changes before an intervention is implemented. Their clinical usefulness will depend on physiological plausibility, transparency, external validation, and demonstrated benefit beyond conventional assessment [106].

**Biological and integrated data sources.** Metabolomics, microbiome profiles, genomics, and additional wearable sensors may refine the characterization of metabolic heterogeneity by identifying contributors that are not directly observable from glucose patterns alone [107]. Integration of these data with CGM may improve phenotype classification and support more individualized assessment [94,110]. Nevertheless, increasing the quantity of data does not necessarily improve clinical meaning. Each additional data source should be judged according to whether it strengthens biological understanding, improves decision-making, or meaningfully changes patient outcomes.

### 8.4. Clinical Workflow Integration

The translation of physiology-informed CGM interpretation into practice will depend on its compatibility with routine clinical workflows. Even a scientifically valid framework is unlikely to influence care if it requires excessive time, fragmented data review, or expertise that is unavailable in typical diabetes settings. Clinical implementation therefore requires practical methods for organizing CGM, dietary, behavioral, medication, and clinical information into a format that supports efficient interpretation [101,112,119].

Clinician training will be essential. Existing CGM education often emphasizes standardized metrics, glucose targets, and medication adjustment. Precision nutrition requires an additional set of competencies, including recognition of recurring glycemic phenotypes, assessment of dietary and behavioral context, evaluation of plausible physiological mechanisms, and communication of uncertainty. Training should also emphasize that a CGM pattern generates hypotheses rather than a definitive mechanistic diagnosis.

Decision support tools may improve consistency while reducing the cognitive burden associated with integrating multiple physiological and contextual data sources [109,110]. Such tools could summarize recurring phenotypes, identify the meals or conditions associated with them, display relevant contextual information, and suggest physiological contributors for clinical consideration. To remain aligned with the framework proposed in this review, decision support should make its interpretive reasoning transparent and preserve clinician oversight rather than provide unexplained dietary directives.

Integration across professional roles is also important. Physicians, diabetes care and education specialists, registered dietitian nutritionists, pharmacists, and other members of the care team may each contribute distinct expertise [120]. A shared interpretive framework could support communication across these disciplines by providing common terminology for describing glycemic phenotypes and their possible contributors.

Patient engagement remains equally important. CGM can make the relationship between daily behaviors and glucose responses more visible, but data presentation alone does not ensure understanding or sustainable change. Patients require guidance in distinguishing meaningful recurring patterns from normal variation and in evaluating glucose responses alongside satiety, nutrient quality, affordability, cultural preferences, treatment burden, and quality of life. Shared decision-making is therefore necessary to ensure that physiology-informed recommendations are both clinically appropriate and practically sustainable.

### 8.5. Future Clinical Implementation

Several developments are needed before physiology-informed precision nutrition can be implemented routinely. First, glycemic phenotypes require clearer operational definitions. Studies differ in how they define postprandial peaks, excursion duration, recovery, baseline elevation, and variability, which limits comparison across populations and technologies [8,50]. Standardized definitions would improve reproducibility and support validation of phenotype-based interpretation.

Second, proposed phenotypes and interpretive pathways should be tested in diverse populations with type 2 diabetes. Validation should include individuals with different disease durations, medication regimens, body compositions, racial and ethnic backgrounds, dietary patterns, socioeconomic circumstances, and levels of glycemic control [2,106]. Phenotype definitions, interpretive frameworks, and predictive models developed in narrowly selected research cohorts may not perform reliably in broader clinical settings.

Third, future trials should evaluate whether phenotype-guided, physiology-informed care improves outcomes beyond CGM feedback or standard nutrition counseling alone. Relevant outcomes extend beyond HbA1c [101] and may include postprandial control, glycemic variability, treatment burden, dietary quality, adherence, patient understanding, cardiometabolic risk, and quality of life. Comparisons should also determine whether the additional complexity and cost of multimodal data integration produce clinically meaningful benefits.

Implementation science will be necessary to establish how the framework can be incorporated into real clinical environments. Research should address staffing, training, visit duration, data interoperability, reimbursement, clinician acceptance, patient usability, and long-term sustainability. The effectiveness of the framework will depend not only on biological validity but also on whether it can be delivered consistently within existing systems of care.

Equitable access must remain a central consideration [112]. CGM, advanced analytics, multi-omics, and digital twin technologies may widen disparities if their benefits are limited to individuals with greater financial resources, digital literacy, specialist access, or technological support. Clinical translation should therefore prioritize scalable approaches that preserve the essential interpretive principles of the framework even when advanced technologies are unavailable. Precision nutrition should reflect greater biological and contextual relevance, not simply greater technological intensity.

The framework proposed in this review does not seek to replace established nutritional guidelines or clinical judgment. Rather, it provides a physiological lens through which CGM-derived glycemic phenotypes can inform individualized nutritional decision-making. Artificial intelligence, digital twins, and emerging biological data may strengthen this process by improving pattern recognition, prediction, and integration across multiple sources of information [94,110]. Their clinical value, however, will depend on whether they support transparent, biologically grounded decisions and lead to feasible, equitable, and meaningful improvements in care.

Ultimately, the value of CGM for precision nutrition lies not in generating more data but in supporting meaningful physiological interpretation. Future implementation should preserve the sequence central to the proposed framework: phenotype recognition, physiological interpretation, individualized strategy, and iterative reassessment.

## 9. Limitations

The framework proposed in this review is intended to support the physiological interpretation of CGM-derived glycemic phenotypes rather than establish definitive biological mechanisms. Although CGM provides continuous insight into glucose dynamics, it does not directly measure the physiological processes responsible for observed glucose patterns. Similar glycemic phenotypes may arise through different combinations of nutrient absorption, hormonal regulation, hepatic glucose production, peripheral glucose disposal, and behavioral influences, while similar physiological disturbances may produce different glucose responses depending on the surrounding metabolic context [1,2]. Consequently, physiology-informed interpretation should be viewed as probabilistic rather than deterministic, providing biologically plausible explanations that inform clinical reasoning rather than definitive mechanistic diagnoses.

The evidence supporting this framework also remains heterogeneous [106]. Studies differ substantially in their populations, dietary interventions, meal standardization, medication use, CGM devices, analytical methods, and reported glycemic outcomes. Furthermore, there is currently no universally accepted system for defining or classifying CGM-derived glycemic phenotypes, limiting direct comparison across studies and reducing the consistency with which findings can be synthesized [2]. Although the available literature supports many of the relationships described throughout this review, continued validation and harmonization of phenotype definitions will be necessary to strengthen their reproducibility and clinical applicability. As a structured narrative review, the framework proposed here is based on critical synthesis and integration of the available evidence rather than a formal quantitative assessment of evidence certainty. Accordingly, it should be viewed as a conceptual model intended to organize current knowledge and guide future investigation rather than establish definitive clinical recommendations.

Application of the framework in clinical practice depends on the availability of sufficient contextual information to support meaningful interpretation. Glucose responses are influenced by factors extending beyond dietary composition, including medication use, physical activity, sleep, psychological stress, illness, and other behavioral or environmental variables that are not fully captured by CGM alone. Likewise, dietary assessment methods may be incomplete or imprecise, limiting confidence in attributing specific glucose patterns to individual nutritional exposures. Consequently, interpretation is only as reliable as the quality and completeness of the accompanying clinical, behavioral, and dietary information.

These limitations do not diminish the potential value of the proposed framework; rather, they define its appropriate scope. The framework should be regarded as an interpretive guide for generating physiologically plausible explanations for observed CGM-derived glycemic phenotypes rather than a diagnostic model capable of inferring definitive mechanisms from glucose data alone. As the evidence base continues to evolve, ongoing validation, refinement, and prospective evaluation will be essential to determine the clinical utility, generalizability, and implementation of physiology-informed CGM interpretation in precision nutrition.

## 10. Future Directions

The framework presented in this review provides a conceptual foundation for physiology-informed interpretation of CGM-derived glycemic phenotypes. Continued progress will depend not only on advances in CGM technology, but also on the development of standardized phenotypes, rigorous validation, clinically meaningful intervention studies, and implementation strategies capable of translating biological understanding into routine diabetes care. The following priorities represent key areas for future investigation.

### 10.1. Validation of Glycemic Phenotypes

A fundamental priority for future research is the validation of the glycemic phenotypes proposed throughout this framework. Future studies should determine whether these phenotypes are reproducible across repeated observations, stable over time, and consistently associated with specific physiological processes in diverse populations with type 2 diabetes [50]. Validation should extend beyond controlled research environments to include individuals with varying disease duration, treatment regimens, metabolic characteristics, and dietary habits. Establishing robust phenotype definitions will provide the foundation upon which physiology-informed CGM interpretation can be refined and applied with greater confidence.

### 10.2. Standardization of CGM-Based Interpretation

Greater standardization is needed to improve the consistency and reproducibility of phenotype-guided interpretation [8]. Harmonized definitions of glycemic phenotypes, standardized reporting of CGM-derived outcomes, and consistent characterization of dietary exposures would facilitate comparison across studies and strengthen evidence synthesis. Development of shared terminology and reporting recommendations may also improve communication among researchers and clinicians while supporting broader implementation of physiology-informed approaches.

### 10.3. Intervention Studies

Although growing evidence supports relationships among dietary exposures, physiology, and CGM responses, prospective intervention studies are needed to determine whether physiology-informed interpretation translates into improved clinical outcomes. Future trials should evaluate whether phenotype-guided nutritional strategies improve glycemic and clinical outcomes compared with approaches based solely on population-level dietary recommendations, carbohydrate counting, or conventional CGM feedback [2,106]. Outcomes should extend beyond HbA1c to include postprandial glucose control, glycemic variability, dietary quality, treatment adherence, quality of life, and long-term cardiometabolic health. Such studies will be essential to establish the clinical effectiveness and practical value of the proposed framework.

### 10.4. Multimodal Data Integration

Future advances in precision nutrition will likely depend on integrating CGM with complementary sources of biological and behavioral information [107]. Dietary intake, physical activity, sleep, medication use, clinical biomarkers, and additional wearable technologies each provide context that may improve understanding of observed glycemic phenotypes. Rather than functioning as an isolated data source, CGM should increasingly be interpreted as one component of an integrated physiological ecosystem in which multiple factors interact to shape glucose regulation. Future research should determine which combinations of data provide meaningful improvements in interpretation while remaining feasible for routine clinical use [94].

### 10.5. Computational Approaches

Advances in artificial intelligence, machine learning, predictive modeling, and digital twin technologies offer opportunities to support physiology-informed interpretation by integrating large and complex datasets [109,110]. These approaches may improve recognition of recurring glycemic phenotypes, identify interactions among dietary and behavioral variables, and assist in generating individualized predictions. However, future computational models should prioritize transparency, interpretability, and physiological plausibility in addition to predictive performance. Technology should enhance biological understanding and clinical decision-making rather than replace the interpretive role of clinicians.

### 10.6. Implementation Science

Successful translation of this framework into routine practice will require evidence beyond biological validity. Future research should evaluate strategies for integrating phenotype-guided interpretation into clinical workflows, including clinician education, decision support systems, interdisciplinary collaboration, reimbursement models, and patient engagement [112]. Implementation studies should also examine scalability across diverse healthcare settings while ensuring equitable access to CGM and related technologies. Ultimately, the long-term impact of physiology-informed precision nutrition will depend not only on advances in science and technology but also on their successful integration into practical, patient-centered diabetes care.

## 11. Conclusions

Type 2 diabetes is characterized by substantial metabolic heterogeneity, resulting in considerable variability in glycemic responses to similar dietary exposures. Continuous glucose monitoring has transformed the ability to observe this variability under free-living conditions, revealing dynamic patterns of glucose regulation that extend beyond conventional measures of glycemic control [8,15]. However, the increasing availability of CGM data has also highlighted an important challenge: quantitative glucose measurements alone provide limited insight into the physiological processes responsible for the observed responses. Interpreting these patterns within an appropriate biological context is therefore essential if CGM is to realize its full potential in supporting individualized nutritional care.

The framework presented in this review provides a physiology-based approach for interpreting CGM-derived glycemic phenotypes by integrating dietary exposures, underlying metabolic physiology, and individual metabolic characteristics. Rather than considering glucose responses as isolated measurements, the framework positions recurring CGM patterns as observable phenotypes that reflect the combined influence of nutrient digestion and absorption, hormonal regulation, hepatic glucose metabolism, peripheral glucose disposal, behavioral factors, and individual biological variability. By linking these processes to characteristic glucose patterns, the framework provides a structured approach for generating physiologically plausible explanations that may support more informed nutritional assessment and decision-making.

The framework also redefines the role of CGM within precision nutrition by emphasizing interpretation rather than measurement alone. Rather than functioning solely as a technology for monitoring glucose concentrations, CGM becomes a tool for understanding how dietary exposures interact with the biological characteristics of the individual. This interpretive perspective shifts emphasis from identifying universally effective dietary strategies toward understanding why individuals respond differently to similar nutritional interventions. Consequently, precision nutrition becomes an iterative process in which recurring glycemic phenotypes are interpreted within their physiological and clinical context, guiding individualized nutritional strategies that can be reassessed and refined as metabolic conditions change.

Although additional validation, standardization, and prospective clinical evaluation remain necessary, the framework presented here provides a conceptual foundation for future research and clinical translation. Continuous glucose monitoring captures the integrated glycemic consequences of dietary exposures, metabolic physiology, behavioral factors, and treatment under free-living conditions rather than directly measuring the underlying physiological mechanisms. By organizing recurring CGM-derived glucose patterns according to plausible physiological contributors, the proposed framework offers a structured, hypothesis-generating approach for individualized nutritional assessment in adults with type 2 diabetes. Continued advances in multimodal data integration and computational approaches may further strengthen this interpretive process, but prospective studies are needed to establish the reproducibility, clinical actionability, and long-term effectiveness of physiology-informed nutritional personalization. Until such evidence is available, the framework is intended to complement established nutritional guidelines and clinical judgment by supporting physiology-informed interpretation of CGM data rather than serving as an independent determinant of dietary recommendations.

## Figures and Tables

**Table 1:** Proposed physiology-based framework for interpreting CGM-derived glycemic phenotypes and their implications for precision nutrition in type 2 diabetes ^a,b^.

Framework Domain	Underlying Physiological Basis	Representative CGM-Derived Glycemic Phenotype	Plausible Physiological and Behavioral Contributors	Implications for Precision Nutrition	Evidence Maturity	Key References
Dietary Exposures	Carbohydrate digestibility, food matrix, starch functionality, fiber content, and nutrient composition influence gastric emptying, intestinal glucose absorption, incretin secretion, and substrate availability.	Sharp early glucose excursion, Prolonged postprandial excursion, reduced excursion amplitude, prolonged return to baseline.	Differences in postprandial curve morphology may reflect variation in carbohydrate absorption kinetics and the interaction between nutrient composition and host physiology rather than carbohydrate quantity alone.	Modification of carbohydrate quality, food matrix, starch functionality, and fiber content may help attenuate unfavorable postprandial glycemic phenotypes in selected individuals.	Established	[2,12,17,19,20]
Meal Structure	Mixed meals, nutrient sequencing, protein and fiber preloads, fat content, meal order, and meal timing modify gastric emptying, incretin responses, insulin secretion, and glucose disposal.	Reduced postprandial incremental area under the curve, Prolonged postprandial excursion, shortened time above range, improved postprandial recovery.	Meal composition and temporal organization may alter the physiological response to identical carbohydrate loads through multiple complementary mechanisms.	Meal sequencing, mixed meal composition, nutrient preloads, and timing strategies may be individualized according to the observed glycemic phenotype and underlying physiology.	Moderate	[10,12,21,22,23]
Individual Metabolic Characteristics	Insulin sensitivity, beta-cell function, hepatic glucose regulation, skeletal muscle glucose uptake, adiposity, body composition, gut microbiome, and genetic or metabolic heterogeneity influence glucose handling.	Significantly different glycemic responses following identical meals between individuals.	Interindividual variability may reflect differences in underlying metabolic physiology rather than dietary exposure alone. Distinct glycemic phenotypes may arise from differing combinations of insulin resistance, beta-cell dysfunction, hepatic glucose dysregulation, body composition, and other sources of metabolic heterogeneity.	Interpretation of CGM responses should incorporate individual metabolic characteristics when developing precision nutrition strategies.	Moderate	[1,2,14,24,25,26]
Chronobiology and Circadian Regulation	Circadian regulation of insulin sensitivity, glucose tolerance, hormonal secretion, and peripheral clock gene activity modifies metabolic responses throughout the day.	Greater postprandial excursions during evening meals, elevated overnight glucose, altered responses according to meal timing or time-restricted eating patterns.	Circadian variation may contribute to temporal differences in glycemic phenotypes that cannot be explained by meal composition alone.	Meal timing and daily carbohydrate distribution may be individualized according to circadian metabolic responses and the observed CGM phenotype.	Emerging	[27,28,29,30,31,32]
Behavioral and Lifestyle Modifiers	Physical activity, sleep quality, sleep consistency, habitual dietary variability, and other lifestyle factors influence insulin action, glucose disposal, and metabolic flexibility.	Elevated day-to-day variability, increased overnight glucose, persistent mean hyperglycemia despite similar average dietary intake.	Behavioral factors may modify physiological responses and contribute to intraindividual variability in CGM-derived glycemic phenotypes.	Lifestyle interventions should be interpreted alongside CGM-derived phenotypes rather than independently of them.	Emerging	[2,33,34,35,36]
CGM-derived Glycemic Phenotypes	CGM integrates the cumulative effects of dietary exposures, physiological regulation, individual metabolic characteristics, medications, and lifestyle factors.	Sharp early excursion; Prolonged postprandial excursion; Persistent daytime hyperglycemia; Elevated overnight baseline; High day-to-day variability.	Sharp early excursion: may reflect rapid carbohydrate absorption, impaired early phase insulin secretion, or other physiological contributors affecting glucose appearance and disposal. Prolonged postprandial excursion: may reflect delayed glucose clearance, impaired peripheral glucose disposal, prolonged gastric emptying, or combinations of these mechanisms. Persistent daytime hyperglycemia: may reflect hepatic insulin resistance, reduced peripheral glucose uptake, impaired insulin secretion, medication effects, or combinations of these influences. Elevated overnight baseline: may reflect increased nocturnal hepatic glucose production, the dawn phenomenon, circadian influences, medication effects, or combinations of these mechanisms. High day-to-day variability: may reflect reduced metabolic flexibility, inconsistent dietary exposure, behavioral factors, medication effects, or combinations of these influences.	CGM-derived phenotypes should be interpreted as integrated physiological manifestations rather than isolated glucose metrics, providing the biological context necessary for individualized nutritional decision-making.	Moderate	[5,6,7,14,15,18]
Advanced Precision Nutrition	Machine learning, multi-omics, digital health platforms, predictive modeling, and integrated physiological data aim to characterize complex metabolic heterogeneity.	Predictive glycemic phenotypes, individualized response profiles, adaptive longitudinal trajectories.	Emerging computational approaches may help translate complex physiological information into individualized metabolic predictions and dynamic nutritional guidance.	Future precision nutrition approaches may combine CGM with biological, behavioral, and computational data to support adaptive and precision nutrition strategies.	Preliminary	[1,2,37,38,39,40]

^a^ Evidence maturity reflects the consistency, quantity, and translational readiness of the available literature rather than a formal certainty assessment. Mechanistic evidence, CGM-based observational evidence, and evidence supporting clinical actionability may differ in maturity. Established evidence is supported by multiple consistent clinical studies and well-characterized physiological mechanisms, whereas emerging and preliminary evidence represent areas requiring further validation before routine clinical implementation. ^b^ The glycemic phenotypes presented in this framework represent proposed, hypothesis-generating interpretive categories intended to facilitate physiology-informed CGM interpretation. Observed CGM patterns should be interpreted in the context of clinical history, dietary exposures, medications, and behavioral factors and should not be regarded as diagnostically specific or formally validated phenotype classifications.

## Data Availability

No new data were created or analyzed in this study. Data sharing is not applicable to this article.

## Abbreviations

The following abbreviations are used in this manuscript:

GIP	Glucose-Dependent Insulinotropic Polypeptide
CGM	Continuous Glucose Monitoring
GLP-1	Glucagon-Like Peptide-1
GLUT4	Glucose Transporter Type 4
HbA1c	Glycated Hemoglobin A1c
TAR	Time Above Range
TBR	Time Below Range
TIR	Time in Range
T2DM	Type 2 Diabetes Mellitus
